# Using a Constraint-Based Method to Identify Chronic Disease Patients Who Are Apt to Obtain Care Mostly Within a Given Health Care System: Retrospective Cohort Study

**DOI:** 10.2196/26314

**Published:** 2021-10-07

**Authors:** Yao Tong, Zachary C Liao, Peter Tarczy-Hornoch, Gang Luo

**Affiliations:** 1 Department of Biomedical Informatics and Medical Education University of Washington Seattle, WA United States; 2 Department of Pediatrics, Division of Neonatology University of Washington Seattle, WA United States; 3 Department of Computer Science and Engineering University of Washington Seattle, WA United States

**Keywords:** asthma, chronic kidney disease, chronic obstructive pulmonary disease, data analysis, diabetes mellitus, emergency department, health care system, inpatients, patient care management

## Abstract

**Background:**

For several major chronic diseases including asthma, chronic obstructive pulmonary disease, chronic kidney disease, and diabetes, a state-of-the-art method to avert poor outcomes is to use predictive models to identify future high-cost patients for preemptive care management interventions. Frequently, an American patient obtains care from multiple health care systems, each managed by a distinct institution. As the patient’s medical data are spread across these health care systems, none has complete medical data for the patient. The task of building models to predict an individual patient’s cost is currently thought to be impractical with incomplete data, which limits the use of care management to improve outcomes. Recently, we developed a constraint-based method to identify patients who are apt to obtain care mostly within a given health care system. Our method was shown to work well for the cohort of all adult patients at the University of Washington Medicine for a 6-month follow-up period. It is unknown how well our method works for patients with various chronic diseases and over follow-up periods of different lengths, and subsequently, whether it is reasonable to perform this predictive modeling task on the subset of patients pinpointed by our method.

**Objective:**

To understand our method’s potential to enable this predictive modeling task on incomplete medical data, this study assesses our method’s performance at the University of Washington Medicine on 5 subgroups of adult patients with major chronic diseases and over follow-up periods of 2 different lengths.

**Methods:**

We used University of Washington Medicine data for all adult patients who obtained care at the University of Washington Medicine in 2018 and PreManage data containing usage information from all hospitals in Washington state in 2019. We evaluated our method’s performance over the follow-up periods of 6 months and 12 months on 5 patient subgroups separately—asthma, chronic kidney disease, type 1 diabetes, type 2 diabetes, and chronic obstructive pulmonary disease.

**Results:**

Our method identified 21.81% (3194/14,644) of University of Washington Medicine adult patients with asthma. Around 66.75% (797/1194) and 67.13% (1997/2975) of their emergency department visits and inpatient stays took place within the University of Washington Medicine system in the subsequent 6 months and in the subsequent 12 months, respectively, approximately double the corresponding percentage for all University of Washington Medicine adult patients with asthma. The performance for adult patients with chronic kidney disease, adult patients with chronic obstructive pulmonary disease, adult patients with type 1 diabetes, and adult patients with type 2 diabetes was reasonably similar to that for adult patients with asthma.

**Conclusions:**

For each of the 5 chronic diseases most relevant to care management, our method can pinpoint a reasonably large subset of patients who are apt to obtain care mostly within the University of Washington Medicine system. This opens the door to building models to predict an individual patient’s cost on incomplete data, which was formerly deemed impractical.

**International Registered Report Identifier (IRRID):**

RR2-10.2196/13783

## Introduction

### Background

Care management is widely used to improve the outcomes of patients with chronic diseases [1]. Typically, a model is built to predict an individual patient’s cost [1-5]. For a patient predicted to incur high costs in the future, we enroll the patient in a care management program for preemptive interventions. Then a care manager will call the patient regularly to check the patient’s status and help arrange health and related services [1-5]. Proper use of care management can lower costs by up to 15%, can reduce hospital visits (emergency department visits and inpatient stays) by up to 40%, and has many other benefits [4,6-13]. Care management is typically used for managing several chronic diseases including asthma, chronic obstructive pulmonary disease, chronic kidney disease, and diabetes, as these diseases fulfill 3 conditions that allow a care management program to be economically feasible for implementation: (1) The disease has a high prevalence rate. (2) If not treated appropriately, the disease can result in acute exacerbations, which are associated with high expenses. (3) Relatively low-cost and effective interventions within the patient’s control are available for the disease [6,14].

In the United States, a patient often obtains care from several health care systems such as academic medical centers and private physician groups. Therefore, the patient’s medical data are spread across these health care systems, and none has complete medical data for the patient. Our prior work [15] showed that less than one-third of hospital visits by adult patients at the University of Washington Medicine (UWM) took place within the UWM in a 6-month follow-up period from April to October 2017. Other researchers showed similar evidence of care fragmentation for adult hospital visits in Massachusetts [16] and for emergency department visits in Indiana [17]. Typical models for forecasting an individual patient’s cost presume complete historical data [14,18,19]. These models cannot be used for a health care system with incomplete data, resulting in many patients with future high costs that could be predicted being missed by care management interventions and having poor outcomes.

Recently, we developed the first constraint-based method to pinpoint a reasonably large subset of patients who are apt to obtain care mostly within a given health care system [15]. For a 6-month follow-up period from April to October 2017, we showed that this constraint-based method worked well for the cohort of all adult patients at the UWM [15]. However, we do not yet know how well our method works for patients with various chronic diseases and over follow-up periods of different lengths. If our method performs well in these cases, for the subset of patients with chronic diseases that is pinpointed by our method and for which the health care system has more complete data, it would then be possible to build a model to predict an individual patient’s cost. This would be better than the current practice of not using any cost prediction model to facilitate care management for this health care system at all.

### Objectives

To understand the potential of our constraint-based method at enabling building models to predict an individual patient’s cost using incomplete medical data, we aimed to assess our method’s performance at the UWM for 5 subgroups of adult patients and over follow-up periods of 2 different lengths. Each subgroup corresponds to one of the 5 major chronic diseases for which care management is used—asthma, chronic kidney disease, chronic obstructive pulmonary disease, type 1 diabetes, and type 2 diabetes.

## Methods

### Ethics Approval

The UWM’s institutional review board approved this retrospective cohort study (STUDY00000118).

### Patient Population

**Figure 1:**
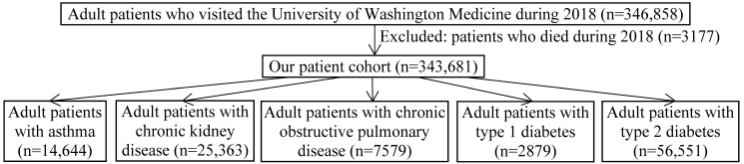
The patient cohort and the 5 patient subgroups.

As the largest academic health care system in Washington state, the UWM provides both clinic-based and hospital-based care for adults. The patient cohort (Figure 1) included adult patients (age ≥18 years) who visited the UWM during 2018 and who had information stored in the UWM’s enterprise data warehouse. Unless explicitly specified as a particular type of visit, a visit can be of any type (outpatient visit, emergency department visit, or inpatient stay) in this paper. Patients who died during 2018 were excluded from the cohort.

### Data Set

**Figure 2:**
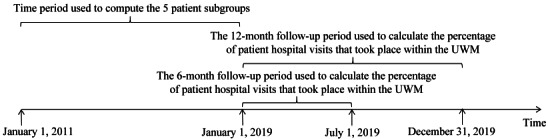
The time periods used to compute the patient subgroups and the percentages of patient hospital visits that took place within the UWM. UWM: University of Washington Medicine.

We used clinical and administrative data for the period from 2011 to 2018 stored in the UWM’s enterprise data warehouse. The data set included information on demographics, visits, diagnoses, laboratory tests, medications, and primary care physicians for patients in our cohort. We also used data of UWM patients from 2019 collected in the commercial product PreManage (Collective Medical Technologies Inc). PreManage contains diagnosis and visit data of hospital visits (emergency department visits and inpatient stays) at all hospitals in Washington state as well as those from many hospitals in other US states [20]. We used January 1, 2019 as the index date to separate the subsequent and prior periods for our analysis task (Figure 2).

### Patient Subgroups

#### Overview

We considered 5 patient subgroups that comprised patients with a specific major chronic disease in our patient cohort in 2018. One subgroup was created for each of 5 major chronic diseases: asthma, chronic kidney disease, chronic obstructive pulmonary disease, type 1 diabetes, and type 2 diabetes.

#### Asthma

A patient was deemed to have asthma in 2018 if the patient had ≥1 International Classification of Diseases, Ninth Revision (ICD-9) or Tenth Revision (ICD-10) diagnosis code for asthma (ICD-9: 493.0x, 493.8x, 493.1x, 493.9x; ICD-10: J45.x) in 2018 [21-23].

#### Chronic Kidney Disease

A patient was deemed to have chronic kidney disease if the patient had an estimated glomerular filtration rate (eGFR) <60 mL/min/1.73m^2^ or proteinuria in 2 measurements that were ≥3 months apart [24,25]. The UWM computes eGFR using the Modification of Diet in Renal Disease equation: eGFR (mL/min/1.73m^2^) = 175 × age^-0.203^ × serum creatinine^-1.154^ × 0.742 (if female) × 1.212 (if Black or African American) [26]. Proteinuria was detected by urine dipstick test result for protein ≥ 1+ (ie, ≥30 mg/dL) [24].

#### Chronic Obstructive Pulmonary Disease

By adjusting the criteria adopted by the National Quality Forum and the Centers for Medicare and Medicaid Services [27-29], we encompassed emergency department and outpatient visit data [30] to identify patients with chronic obstructive pulmonary disease. A patient was deemed to have chronic obstructive pulmonary disease if the patient was ≥40 years and fulfilled any of these 4 conditions: (1) an outpatient visit diagnosis code of chronic obstructive pulmonary disease (ICD-9: 491.22, 491.21, 491.9, 491.8, 493.2x, 492.8, 496; ICD-10: J42, J41.8, J44.*, J43.*) followed by ≥1 prescription of long-acting muscarinic antagonist (aclidinium, glycopyrrolate, tiotropium, and umeclidinium) within 6 months, (2) ≥1 emergency department or ≥2 outpatient visit diagnosis codes of chronic obstructive pulmonary disease (ICD-9: 491.22, 491.21, 491.9, 491.8, 493.2x, 492.8, 496; ICD-10: J42, J41.8, J44.*, J43.*), (3) ≥1 inpatient stay discharge having a principal diagnosis code of chronic obstructive pulmonary disease (ICD-9: 491.22, 491.21, 491.9, 491.8, 493.2x, 492.8, 496; ICD-10: J42, J41.8, J44.*, J43.*), and (4) ≥1 inpatient stay discharge having a principal diagnosis code of respiratory failure (ICD-9: 518.82, 518.81, 799.1, 518.84; ICD-10: J96.0*, J80, J96.9*, J96.2*, R09.2) and a secondary diagnosis code of acute chronic obstructive pulmonary disease exacerbation (ICD-9: 491.22, 491.21, 493.22, 493.21; ICD-10: J44.1, J44.0).

#### Type 1 and Type 2 Diabetes

We used Nichols et al’s method [31] to identify patients with diabetes. A patient was deemed to have diabetes if the patient had ≥1 inpatient stay diagnosis code for diabetes (ICD-9: 250.x, 357.2, 362.0x, 366.41; ICD-10: E10.x, E11.x) or if any 2 of the following events occurred on the patient within 2 years of each other: (1) hemoglobin A_1c_ (HbA_1c_) ≥6.5%, (2) random plasma glucose ≥200 mg/dL, (3) fasting plasma glucose ≥126 mg/dL, (4) an outpatient visit diagnosis code of diabetes (ICD-9: 250.x, 357.2, 362.0x, 366.41; ICD-10: E10.x, E11.x), and (5) a prescription of antihyperglycemic medication (α-glucosidase inhibitor, amylin analogue, biguanide, dipeptidyl peptidase-4 inhibitor, incretin mimetic, insulin, meglitinide, sulfonylurea, and thiazolidinedione). Two events of the same type, such as 2 instances of HbA_1c_ ≥6.5%, qualified as separate events if they occurred on 2 different days. We did not count 2 prescriptions of metformin or thiazolidinedione with no other manifestation of diabetes, as metformin (a biguanide) and thiazolidinedione could be used for other diseases. We also excluded events that occurred during a pregnancy.

We used Klompas et al’s method [32,33] to distinguish type 1 diabetes and type 2 diabetes. Using all diagnosis codes, laboratory test results, and medication prescriptions during the period 2011 to 2018, we deemed a patient with diabetes to have type 1 diabetes if the patient fulfilled any of the following 4 conditions: (1) the number of type 1 diabetes diagnosis codes (ICD-9: 250.x3, 250.x1; ICD-10: E10.x) was greater than the number of type 2 diabetes diagnosis codes (ICD-9: 250.x2, 250.x0; ICD-10: E11.x) and there was a prescription of glucagon, (2) the number of type 1 diabetes diagnosis codes (ICD-9: 250.x3, 250.x1; ICD-10: E10.x) was greater than the number of type 2 diabetes diagnosis codes (ICD-9: 250.x2, 250.x0; ICD-10: E11.x) and there were no prescriptions of oral hypoglycemic medications other than metformin, (3) a negative C-peptide laboratory test result, and (4) a positive diabetes autoantibody laboratory test result. A patient with diabetes was deemed to have type 2 diabetes if the patient was not deemed to have type 1 diabetes.

### Constraint-Based Method for Identifying Patients

We looked at 3 UWM hospitals whose clinical and administrative data are kept in the UWM’s enterprise data warehouse: University of Washington Medical Center, Harborview Medical Center, and Northwest Hospital (all are in Seattle, Washington). To identify patients who are apt to obtain care mostly within the UWM, we used the parameterized primary care physician constraint developed in our recent paper [15]: the patient has a UWM primary care physician and resides within *d* km of at least 1 of the 3 UWM hospitals. The distance between a UWM hospital and a patient’s home is the ellipsoid great-circle distance computed by the distVincentyEllipsoid function contained in R’s geosphere package (version 1.5-5 [34]). *d* is a parameter. For all UWM adult patients and the follow-up period of 6 months, we showed that the optimal value of *d* is approximately 8 km (5 miles) [15].

### Data Analysis

We considered 2 follow-up periods: the subsequent 6 months (January 1, 2019 to June 30, 2019) and the subsequent 12 months (January 1, 2019 to December 31, 2019) (Figure 2). The 6-month follow-up period was chosen to be consistent with the duration of the follow-up period used in our prior paper [15]. The 12-month follow-up period was chosen because, to facilitate care management, typically a minimum of 1 year of historical data is needed to build models that predict an individual patient’s cost [14]. For each of the 5 patient subgroups and each of the 2 follow-up periods, we computed our method’s performance in identifying patients who are apt to obtain care mostly within the UWM. We employed administrative data in the UWM’s enterprise data warehouse to assess whether a patient fulfilled the parameterized primary care physician constraint. For each of the 5 patient subgroups, we calculated the percentage of patients identified by the constraint = *n_0_*/*m_0_*×100%. Here, *n_0_* is the number of patients in the subgroup fulfilling the constraint. *m_0_* is the number of patients in the subgroup. For all patients in the subgroup fulfilling the constraint, we used PreManage data to calculate

1. the percentage of their hospital visits taking place within the UWM in the subsequent 6 months = *n_1_*/*m_1_*×100%, where *n_1_* is the number of their hospital visits taking place within the UWM in the subsequent 6 months, and *m_1_* is the number of their hospital visits taking place anywhere in the subsequent 6 months; and
2. the percentage of their hospital visits taking place within the UWM in the subsequent 12 months = *n_2_*/*m_2_*×100%, where *n_2_* is the number of their hospital visits taking place within the UWM in the subsequent 12 months, and *m_2_* is the number of their hospital visits taking place anywhere in the subsequent 12 months.

Since an average hospital visit costs much more than an average visit of another type, this percentage signifies the proportion of those patients’ care obtained from the UWM.

To determine the optimal value of the distance threshold parameter *d*, we balanced 2 goals. (1) The proportion of hospital visits taking place within the UWM should be as large as possible for patients fulfilling the constraint. This will maximize the completeness of UWM medical data and minimize bias in the results of analyses done on those data. As outpatient visits are often handled by primary care physicians and these patients each have a UWM primary care physician, we expect most of their outpatient visits to occur within the UWM in the subsequent 12 months. (2) The percentage of patients fulfilling the constraint should be as large as possible. This will help maximize the impact of the application using UWM medical data.

To show how our method performs for every UWM hospital, for all patients in the subgroup fulfilling the constraint, we employed PreManage data to calculate (1) the percentage of their hospital visits taking place at the UWM hospital in the subsequent 6 months = *n_3_*/*m_1_*×100%, where *n_3_* is the number of their hospital visits taking place at the UWM hospital in the subsequent 6 months, and *m_1_* is the number of their hospital visits taking place anywhere in the subsequent 6 months, and (2) the percentage of their hospital visits taking place at the UWM hospital in the subsequent 12 months = *n_4_*/*m_2_*×100%, where *n_4_* is the number of their hospital visits taking place at the UWM hospital in the subsequent 12 months, and *m_2_* is the number of their hospital visits taking place anywhere in the subsequent 12 months.

## Results

The cohort of adult patients who visited UWM facilities during 2018 with information stored in the UWM’s enterprise data warehouse comprised 343,681 patients (Table 1).

**Figure 3:**
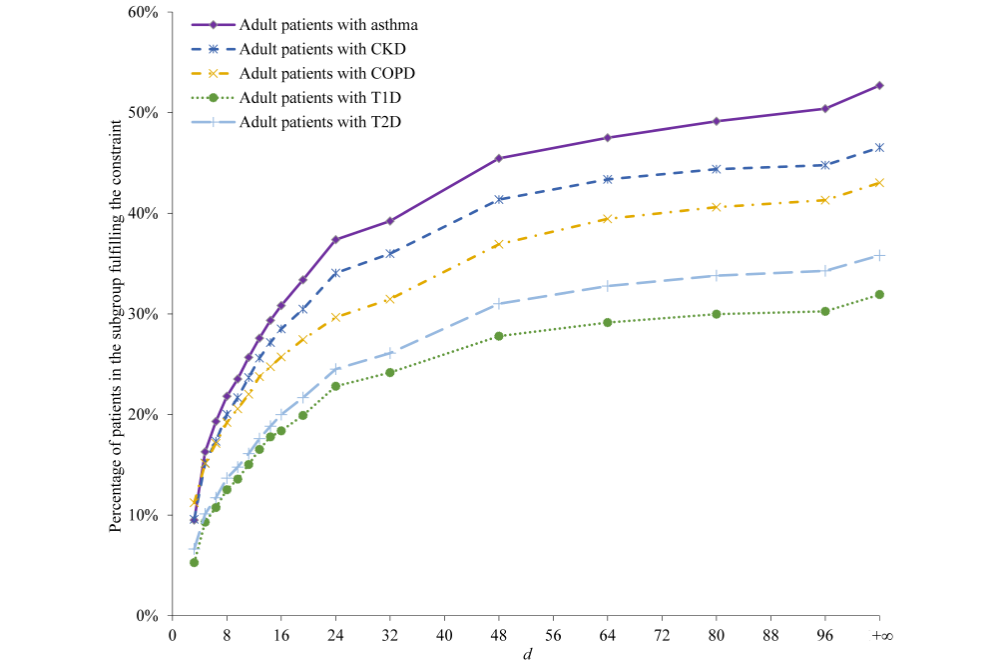
The percentage of patients in each of the 5 patient subgroups fulfilling the parameterized primary care physician constraint. CKD: chronic kidney disease. COPD: chronic obstructive pulmonary disease. T1D: type 1 diabetes. T2D: type 2 diabetes.

**Figure 4:**
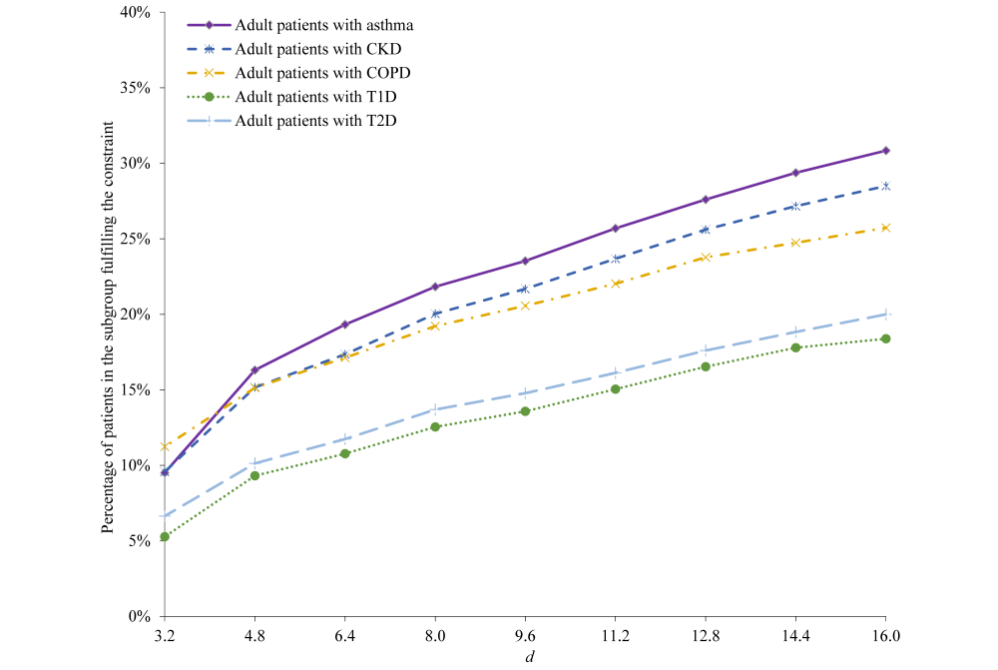
The percentage of patients in each of the 5 patient subgroups fulfilling the parameterized primary care physician constraint, when *d* is ≤10. CKD: chronic kidney disease. COPD: chronic obstructive pulmonary disease. T1D: type 1 diabetes. T2D: type 2 diabetes.

Figures 3 and 4 present the percentage of patients fulfilling the parameterized primary care physician constraint for each of the 5 patient subgroups. The percentage rises with increase in *d*, at first swiftly when *d* is small and then at a slower pace when *d* grows larger.

**Table 1:** Demographic and clinical characteristics of the study cohort.

Characteristic			Patients (n=343,681), n (%)
**Age**			
	18 to <40 years	120,422 (35.04)	
	40 to 65 years	149,418 (43.48)	
	>65 years	73,841 (21.49)	
**Gender**			
	Male	159,964 (46.54)	
	Female	183,701 (53.45)	
	Unknown or not reported	16 (<.01)	
**Race**			
	Black or African American	25,513 (7.42)	
	American Indian or Alaska native	4795 (1.40)	
	Asian	34,474 (10.03)	
	Native Hawaiian or other Pacific islander	2843 (0.83)	
	Multiple races	1 (<0.01)	
	Unknown or not reported	45,094 (13.12)	
	White	230,961 (67.20)	
**Ethnicity**			
	Non-Hispanic	271,582 (79.02)	
	Hispanic	21,718 (6.32)	
	Unknown or not reported	50,381 (14.66)	
**Insurance**			
	Private	163,908 (47.69)	
	Public (Medicare and Medicaid)	160,026 (46.56)	
	Self-paid or charity	19,747 (5.75)	
**Disease**			
	Asthma	14,644 (4.26)	
	Chronic kidney disease	25,363 (7.38)	
	Chronic obstructive pulmonary disease	7579 (2.21)	
	Type 2 diabetes	56,551 (16.45)	
	Type 1 diabetes	2879 (0.84)	

**Figure 5:**
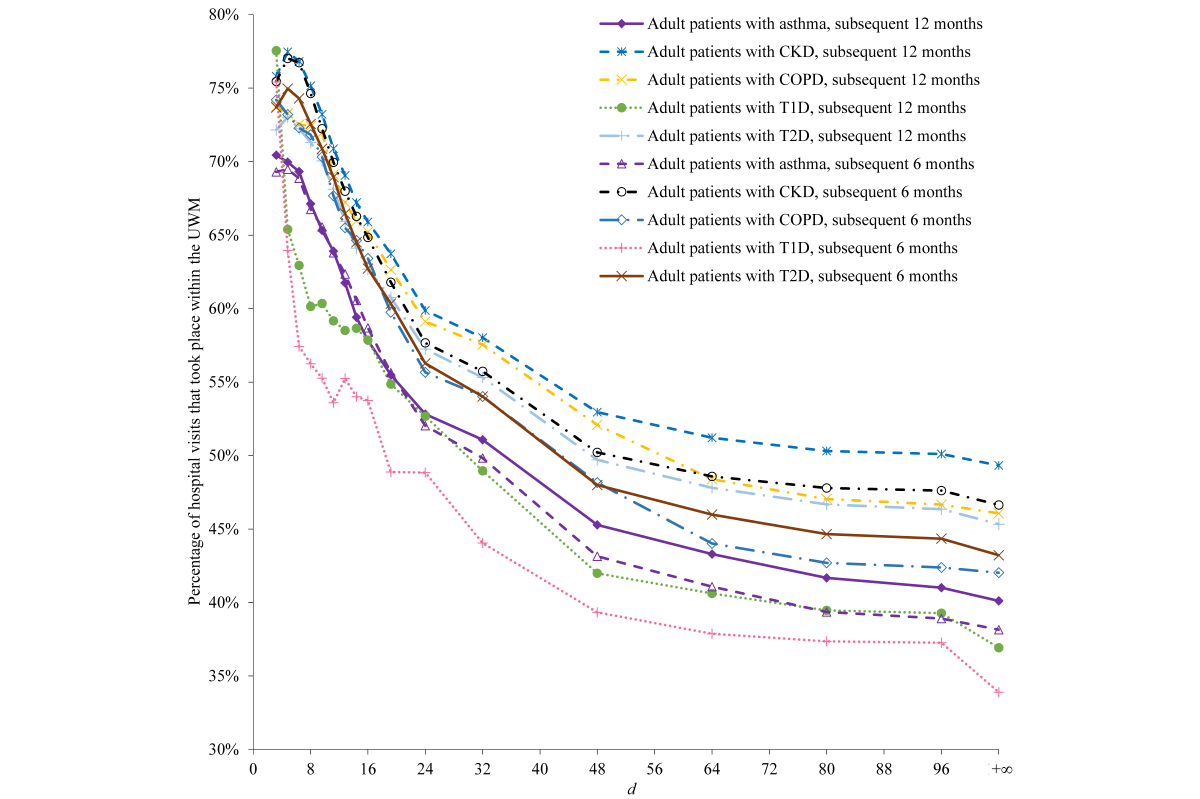
For all patients in each of the 5 patient subgroups fulfilling the parameterized primary care physician constraint, the percentages of their hospital visits taking place within the University of Washington Medicine (UWM) in the subsequent 6 months and in the subsequent 12 months. CKD: chronic kidney disease. COPD: chronic obstructive pulmonary disease. T1D: type 1 diabetes. T2D: type 2 diabetes.

**Figure 6:**
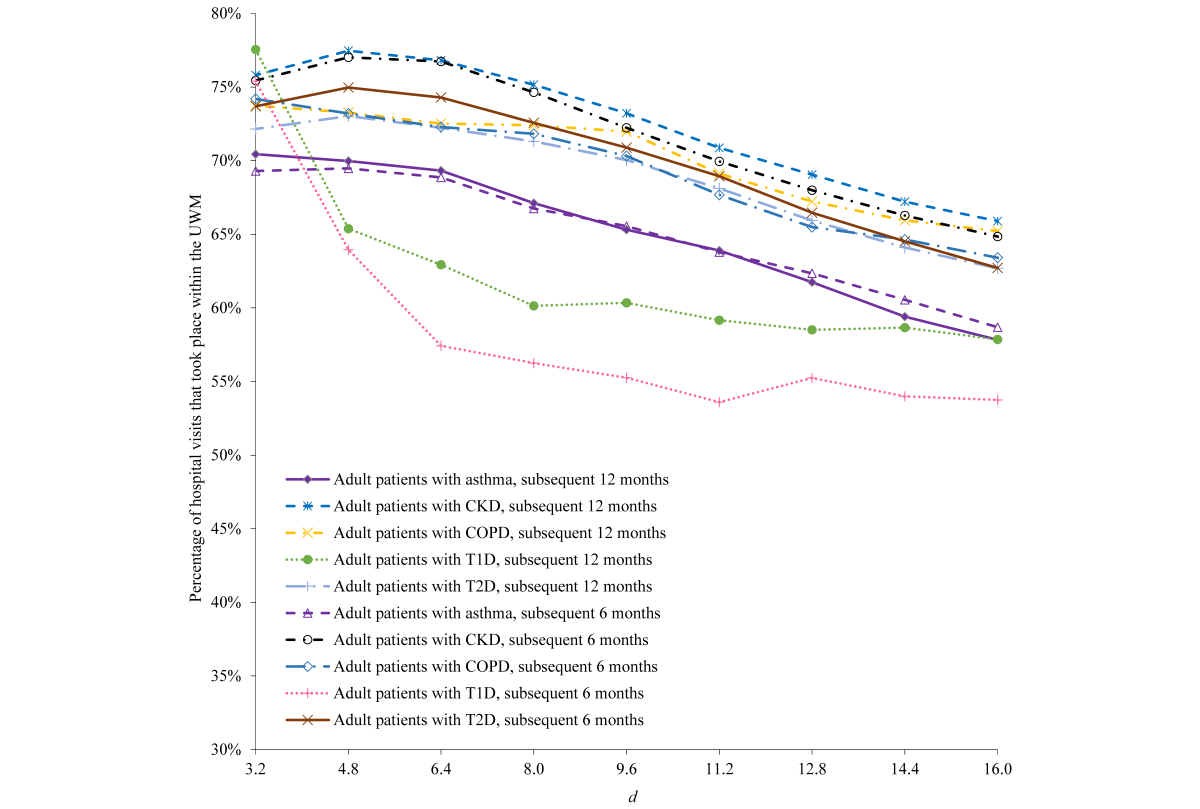
For all patients in each of the 5 patient subgroups fulfilling the parameterized primary care physician constraint when *d* is ≤10, the percentages of their hospital visits taking place within the University of Washington Medicine (UWM) in the subsequent 6 months and in the subsequent 12 months. CKD: chronic kidney disease. COPD: chronic obstructive pulmonary disease. T1D: type 1 diabetes. T2D: type 2 diabetes.

For all patients in each of the 5 patient subgroups fulfilling the parameterized primary care physician constraint, Figures 5 and 6 present the percentages of their hospital visits taking place within the UWM in the subsequent 6 months and in the subsequent 12 months. Except for a few cases at small values of *d*, the percentage decreases with increasing *d*, swiftly when *d* is small and then slowly when *d* is large.

We chose *d*=8 km as the optimal value to use for each patient subgroup and each follow-up period. Table 2 shows the corresponding performance measures of our constraint-based method.

For every UWM hospital and all patients in each of the 5 patient subgroups fulfilling the parameterized primary care physician constraint, Multimedia Appendix 1 shows the percentages of their hospital visits taking place at the UWM hospital in the subsequent 6 months and in the subsequent 12 months.

**Table 2:** For *d*=8 km and for each of the 5 patient subgroups, the percentage of patients fulfilling the parameterized primary care physician constraint, the percentages of patient hospital visits taking place at the University of Washington Medicine (UWM) in the subsequent 6 months and in the subsequent 12 months, and the percentages of hospital visits by patients fulfilling the constraint that took place within the UWM in the subsequent 6 months and in the subsequent 12 months.

Patient subgroup	Patients fulfilling the parameterized primary care physician constraint, n/N (%)	Patient hospital visits taking place within the UWM^a^ in the subsequent 6 months, n/N (%)	Hospital visits by patients fulfilling the constraint that took place within the UWM in the subsequent 6 months, n/N (%)	Patient hospital visits taking place within the UWM in the subsequent 12 months, n/N (%)	Hospital visits by patients fulfilling the constraint that took place within the UWM in the subsequent 12 months, n/N (%)
Adult patients with asthma	3194/14,640 (21.81)	2648/7135 (37.11)	797/1194 (66.75)	6857/18,206 (37.66)	1997/2975 (67.13)
Adult patients with chronic kidney disease	5081/25,363 (20.03)	7503/18,404 (40.77)	2178/2918 (74.64)	19,558/45,994 (42.52)	5634/7496 (75.16)
Adult patients with chronic obstructive pulmonary disease	1456/7579 (19.21)	2587/6659 (38.85)	831/1157 (71.82)	7026/16,941 (41.47)	2179/3009 (72.42)
Adult patients with type 1 diabetes	361/2879 (12.54)	317/1333 (23.78)	63/112 (56.25)	845/3330 (25.38)	169/281 (60.14)
Adult patients with type 2 diabetes	7744/56,551 (13.69)	10,926/30,707 (35.58)	2847/3923 (72.57)	29,272/79,775 (36.69)	7177/10,065 (71.31)

^a^UWM: University of Washington Medicine.

## Discussion

### Principal Results

For each of the 5 major chronic diseases most relevant to care management (asthma, chronic obstructive pulmonary disease, chronic kidney disease, type 1 diabetes, and type 2 diabetes), our constraint-based method with a properly chosen value of the parameter *d* can pinpoint a reasonably large subset of patients who are apt to obtain care mostly within the UWM. Using our method to pinpoint a subset of UWM adult patients with asthma, we roughly doubled the percentage of patient hospital visits taking place within the UWM in the subsequent 6 months from 37.11% (2648/7135) to 66.75% (797/1194), and the corresponding percentage for the subsequent 12 months from 37.66% (6857/18,206) to 67.13% (1997/2975). The results for adult patients with chronic kidney disease, adult patients with chronic obstructive pulmonary disease, adult patients with type 1 diabetes, and adult patients with type 2 diabetes are relatively similar. As the patients fulfilling the constraint all have a UWM primary care physician, we expect a majority of their outpatient visits to happen within the UWM in the subsequent 12 months, although we did not examine this in our study.

### Explanation of the Results Shown in Figure 5

UWM primary care physicians are inclined to refer within the UWM. Thus, intuitively, patients with a UWM primary care physician are apt to obtain a larger percentage of their care from the UWM than other patients. All else being equal, the UWM tends to provide a larger portion of a patient’s care when the patient resides closer to UWM hospitals. This is reflected in Figure 5. When *d*=+, distance is no longer relevant for identifying patients. No matter how small *d* is, for all patients in each of the 5 patient subgroups fulfilling the parameterized primary care physician constraint, the percentage of their hospital visits taking place within the UWM in the subsequent 6 months (or in the subsequent 12 months) never becomes 100%, partially because patients can also visit multiple non-UWM hospitals within 1.6 km of some of the UWM hospitals. For each positive *d*, the percentage is relatively similar across the 5 patient subgroups and the 2 follow-up periods.

### Comparison With our Prior Work

The findings in this paper are relatively similar to those of our previous study [15]—for the group of all adult patients and the 6-month follow-up period from April to October 2017. The optimal value of *d*=8 km found in this study is the same as that chosen in our previous study. In our previous study, using our constraint-based method with a parameter value of *d*=8 km to pinpoint 16.01% (55,707/348,054) of the UWM adult patients, we roughly doubled the percentage of patient hospital visits taking place within the UWM in the subsequent 6 months from 31.80% (39,171/123,162) to 69.38% (10,501/15,135).

### Differences in the Results for Patients With Type 1 Diabetes and Patients With Type 2 Diabetes

Type 1 diabetes tends to occur in younger people than type 2 diabetes. There are many young adults who are students at the University of Washington and several other universities in the Seattle metropolitan area. During the summer and other university breaks, many of these students return to their hometowns outside the Seattle metropolitan area that the UWM primarily serves. The hospital visits that they incur during these periods are likely to be outside of the UWM system. Partly due to this, as shown in Table 2, the percentage of hospital visits by UWM adult patients with type 1 diabetes that took place within the UWM in each follow-up period is approximately 30% less than the corresponding percentage for UWM adult patients with type 2 diabetes. For patients fulfilling the parameterized primary care physician constraint with *d*=8 km (5 miles), the percentage of hospital visits by patients with type 1 diabetes taking place within the UWM in each follow-up period ranges from 15% to 30% less than the corresponding percentage for patients with type 2 diabetes.

### Possible Use of our Results

We showed that for each of 5 major chronic diseases most relevant to care management, the UWM offers most of the care and has decently complete medical data for patients fulfilling the parameterized primary care physician constraint with *d*=8 km (5 miles). For these patients, we can build a predictive model to identify future high-cost patients and intervene preemptively via care management to avert poor outcomes [1-5]. As patients residing farther from the 3 UWM hospitals were inclined to obtain a smaller percentage of their care from the UWM, the UWM could consider adopting differing preventive interventions for patients residing at different distances from the UWM hospitals, which could help care management gain better results. For patients obtaining only a small percentage of their care from the UWM, it is hard for the UWM to adopt costly preventive interventions in an economic way.

### Possible Ways to Assess our Method’s Performance for Other Health Care Systems That Have No Access to PreManage Data

Like many other health care systems, the UWM has no complete claims data on its patients’ health care use outside of the UWM. If a health care system has complete claims data on its patients’ outside health care use, we could employ claims data instead of PreManage data to perform a similar study.

A health care system with no access to PreManage data could also adopt our method. Without using PreManage data, one could assess our method’s performance by asking some patients of the health care system about care obtained elsewhere.

### Limitations

This study has 2 limitations, which could be interesting topics for future work.

This study assessed the performance of our constraint-based method for 5 chronic diseases at the UWM, which primarily serves an urban region. To know how well our method generalizes to other health care systems, we need to redo our analysis at other health care systems, such as those providing care to rural regions or primarily serving urban regions. Residence are more concentrated in urban regions than in rural regions. For a health care system primarily serving rural regions, we expect *d*>8 km for the optimal value.

For a health care system having incomplete medical data for its patients, we can employ our method to identify a subset of patients who are apt to obtain care mostly within the health care system and assess the degree of data incompleteness for this subset. Analyzing incomplete data could lead to biased results, which are better than no result if the degree of bias is small. At present, we know neither the exact relationship between data incompleteness and bias nor the extent of data incompleteness that can be tolerated before the results of data analysis become invalid. This is a gap. To assess whether our method could safely enable the data analysis task in such a health care system, we could obtain a more complete data set from Kaiser Permanente or any other similar healthcare system, remove different portions of the data set, and assess the effect on analysis results.

### Conclusions

Our constraint-based method to pinpoint a reasonably large subset of patients who are apt to obtain care mostly within a given health care system opens the door to building models to predict an individual patient’s cost on incomplete data, which was formerly deemed infeasible.

## Appendix

Multimedia Appendix 1 Results for every University of Washington Medicine hospital.

## Abbreviations

eGFR: estimated glomerular filtration rate
HbA_1c_: hemoglobin A_1c_
ICD-9: International Classification of Diseases, Ninth Revision
ICD-10: International Classification of Diseases, Tenth Revision
UWM: University of Washington Medicine
