# Supplementary material for: Using a Constraint-Based Method to Identify Chronic Disease Patients Who Are Apt to Obtain Care Mostly Within a Given Health Care System: Retrospective Cohort Study
Source: JMIR Form Res. 2021 Oct 7;5(10):e26314. doi: 10.2196/26314 (PMC8532011; doi:10.2196/26314)
Supplement: Multimedia Appendix 1 [file formative_v5i10e26314_app1.pdf]

Appendix

For every UWM hospital and all patients in each of the 5 patient subgroups fulfilling the parameterized primary care physician constraint, Figures 7-11 show the percentages of their hospital visits taking place at the UWM hospital in the subsequent 6 months and in the subsequent 12 months. For every (patient subgroup, positive *d*) pair, each percentage differs across the 3 UWM hospitals. As *d* grows, each percentage drops at similar paces across the 3 UWM hospitals. For each (positive *d*, UWM hospital) pair, the percentage is relatively similar across the 5 patient subgroups and the 2 follow-up periods.

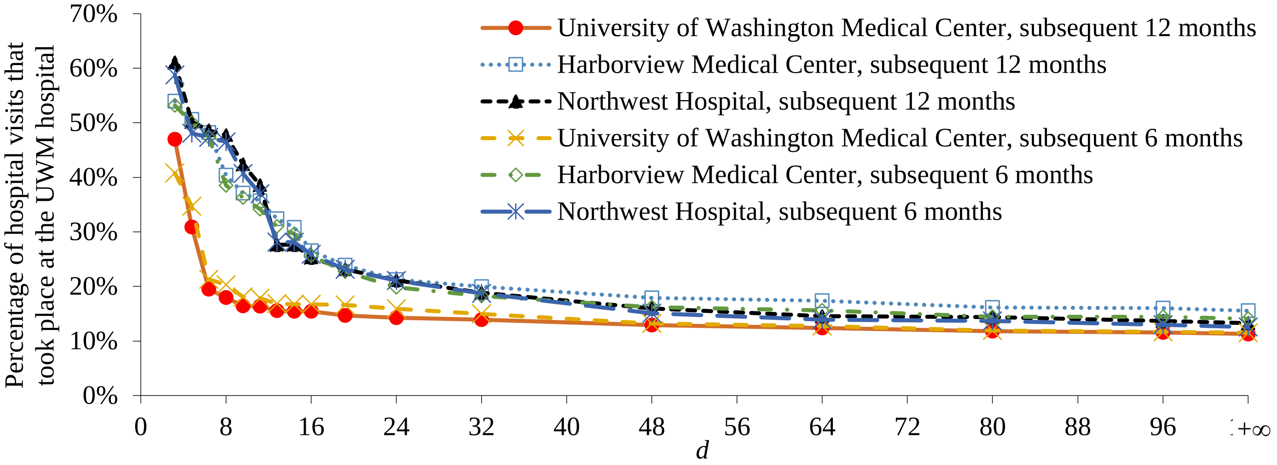

Figure 7. For every University of Washington Medicine (UWM) hospital and all adult patients with asthma fulfilling the parameterized primary care physician (PCP) constraint, the percentages of their hospital visits taking place at the UWM hospital in the subsequent 6 months and in the subsequent 12 months.

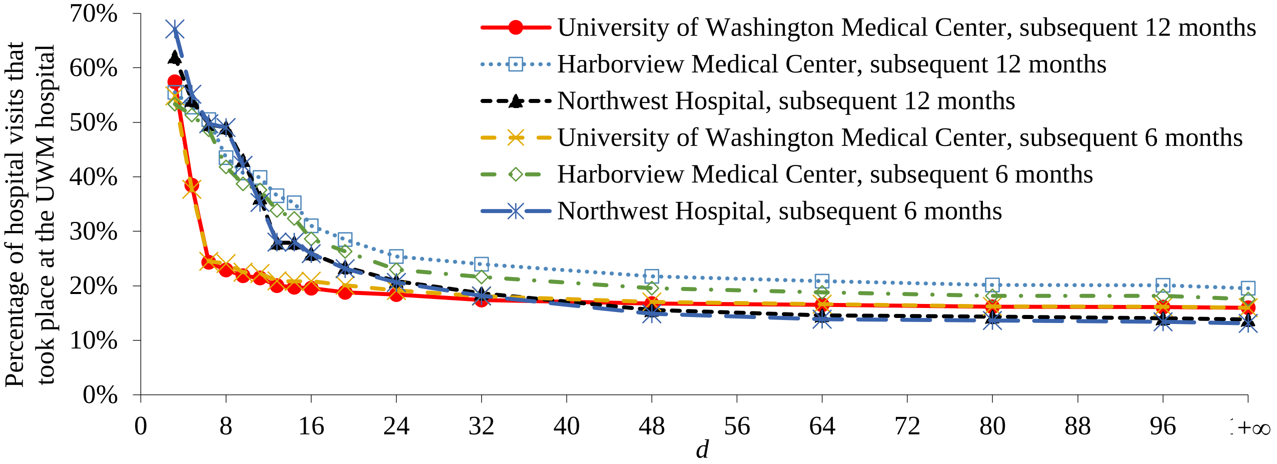

Figure 8. For every University of Washington Medicine (UWM) hospital and all adult patients with chronic kidney disease fulfilling the parameterized primary care physician (PCP) constraint, the percentages of their hospital visits taking place at the UWM hospital in the subsequent 6 months and in the subsequent 12 months.

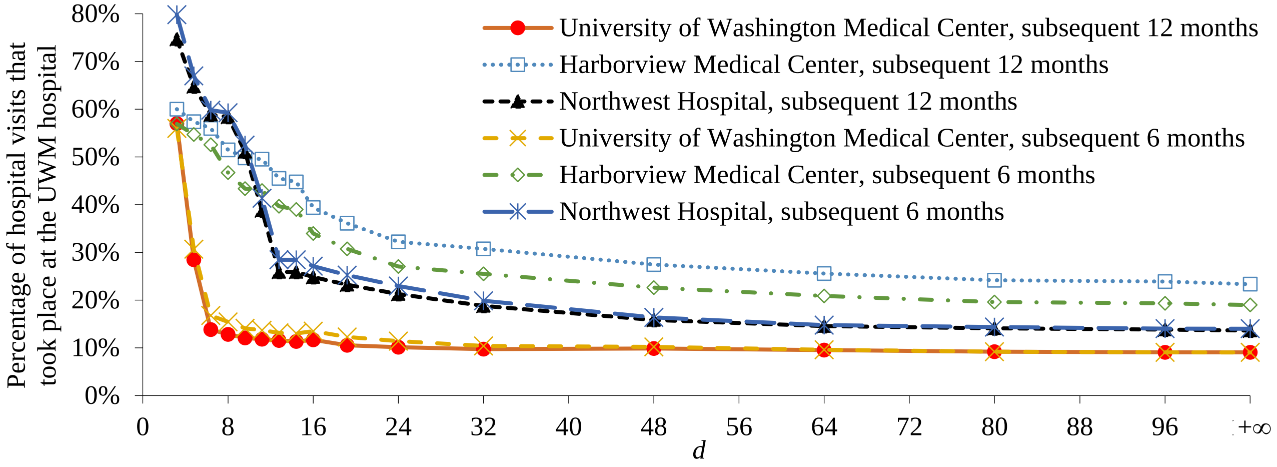

Figure 9. For every University of Washington Medicine (UWM) hospital and all adult patients with chronic obstructive pulmonary disease fulfilling the parameterized primary care physician (PCP) constraint, the percentages of their hospital visits taking place at the UWM hospital in the subsequent 6 months and in the subsequent 12 months.

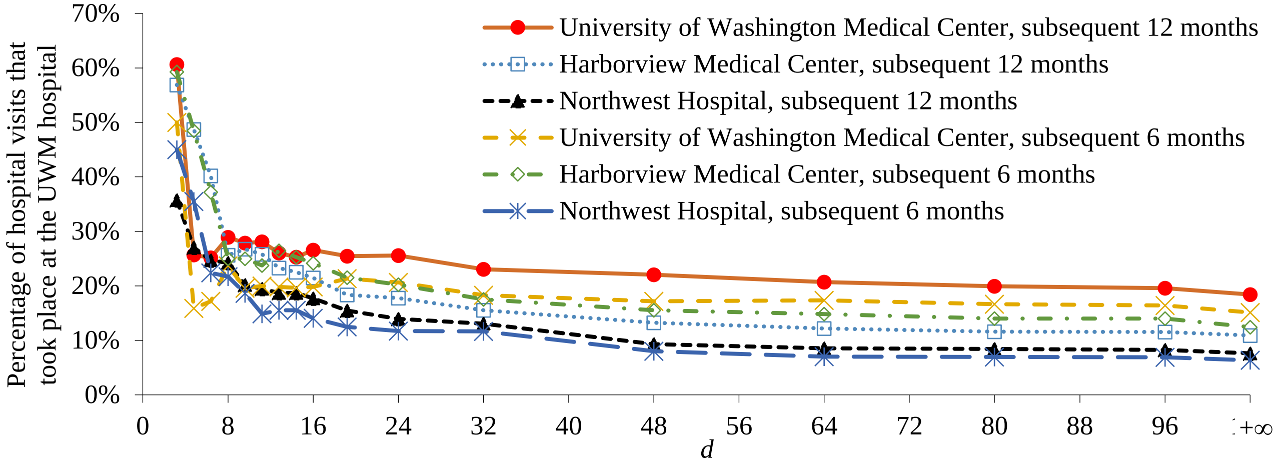

Figure 10. For every University of Washington Medicine (UWM) hospital and all adult patients with type 1 diabetes fulfilling the parameterized primary care physician (PCP) constraint, the percentages of their hospital visits taking place at the UWM hospital in the subsequent 6 months and in the subsequent 12 months.

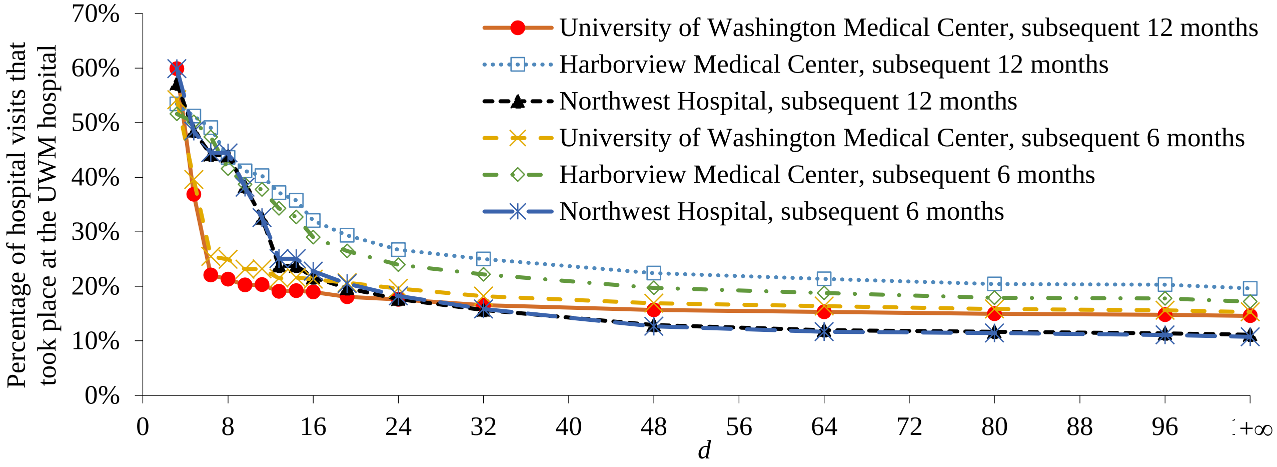

Figure 11. For every University of Washington Medicine (UWM) hospital and all adult patients with type 2 diabetes fulfilling the parameterized primary care physician (PCP) constraint, the percentages of their hospital visits taking place at the UWM hospital in the subsequent 6 months and in the subsequent 12 months.
